# Primary ovarian adenosarcoma with elevated Ca-125 levels and normal ascitic fluid cytology: a case report and review of literature

**DOI:** 10.3332/ecancer.2012.284

**Published:** 2012-12-17

**Authors:** PN Shakuntala, K Umadevi, A Usha, N Abhilasha, UD Bafna

**Affiliations:** 1 Department of Gynaecologic Oncology, Kidwai Memorial Institute of Oncology, Dr. M.H. Mari Gowda Road, Bengaluru 560029, Karnataka, India; 2 Department of Pathology, Kidwai Memorial Institute of Oncology, Dr. M.H. Mari Gowda Road, Bengaluru 560029, Karnataka, India

**Keywords:** adenosarcoma ovary, CA-125, ascitic fluid cytology, chemotherapy

## Abstract

**Objective:**

Ovarian adenosarcoma is a very rare tumour for which treatment options vary. We will consider the option of optimal cytoreductive surgery followed by adjuvant chemotherapy consisting of ifosamide (mesna) and adriamycin to prevent systemic metastasis, and will observe the role of serial CA-125 levels as a follow-up marker.

**Case report:**

We report a case of ovarian adenosarcoma in a 38-year-old woman presenting with abdominal pain, distention due to massive ascites. She had undergone total abdominal hysterectomy 8 months previously for abnormal uterine bleeding. She underwent paracentesis followed by optimal cytoreductive surgery. A post-operative histopathologic diagnosis of primary adenosarcoma was confirmed. She was assigned a stage III C cancer. She received five cycles of ifosamise (mesna) and adriamycin and is on follow-up with serial CA-125 levels. There is no evidence of recurrence clinically, biochemically, or radiologically for more than 12 months.

**Conclusion:**

Multimodality treatment comprising optimal cytoreductive surgery followed by ifosamide (mesna) and adriamycin-based chemotherapy may be an option for treatment of these aggressive tumours. Follow-up with serial CA-125 values in advanced stage adenosarcoma of the ovary is a novel observation which needs to be researched.

## Introduction

Mullerian adenosarcoma is very rare. In 1974, Clement and Scully described uterine adenosarcomas for the first time [1], since then there have been 60 cases of ovarian adenosarcomas described in the literature [2–10], and only four cases of ovarian adenosarcoma with elevated levels of CA-125 have been reported [3–5, 8]. Most of the cases reported have associated endometriosis or an adenosarcoma arising from an endometriotic area, but the direct relation between this tumour and endometriosis has not been made clear in the literature [7, 10]. The present case was pure or homologous adenosarcoma of the ovary without associated endometriosis and an elevated CA-125 level. Adenosarcomas contain benign Müllerian type glands and generally low-grade sarcomatous stroma that resembles stromal sarcoma [9]. The recommended treatment of adenosarcoma is optimal cytoreduction followed by adjuvant therapy for high-grade sarcomatous component if present [4, 7]. Various treatment options ranging from oral progesterone [8], fertility sparing surgery to optimal cytoreductive surgery [4, 7], post-operative radiation therapy and adjuvant chemotherapy [2] have been described in the literature.

## Case report

**Figure 1:**
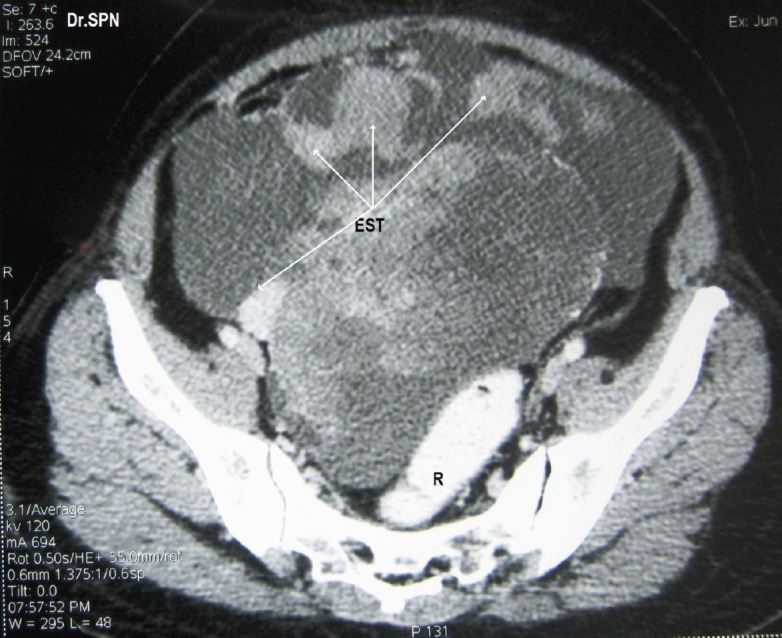
CT scan with oral and rectal contrast (R) showing a pelvic mass with multiple heterogeneously enhancing soft tissue masses with central necrotic areas within pelvis not separately seen from the ovaries (EST).

**Figure 2:**
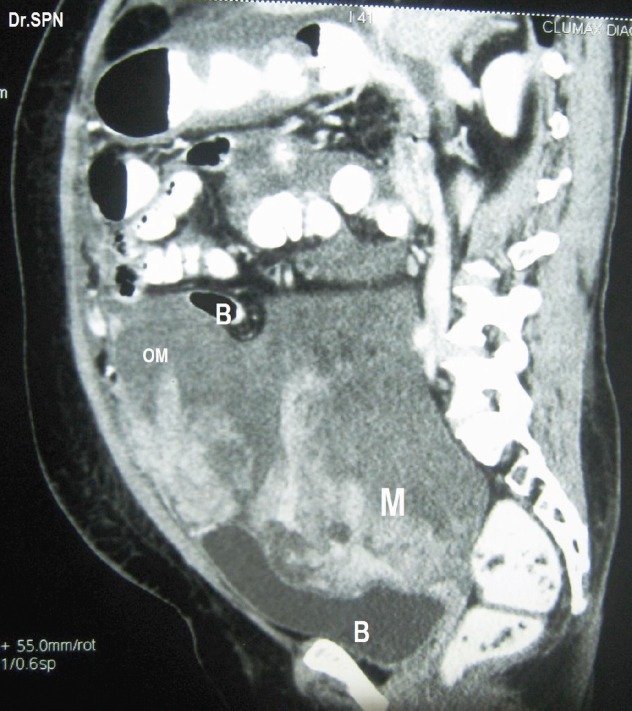
Sagittal section showing heterogeneously enhancing soft tissue lesions within omentum (OM), M- pelvic mass seen indenting bladder base (B), Bowel (B) loops are displaced upwards.

A 38-year-old multiparous woman presented with history of abdominal distension and pain for almost 3 weeks. She had undergone hysterectomy 8 months previously for abnormal uterine bleeding. Following abdominal paracentesis for massive ascites, a mobile mass was palpated measuring about 12 × 10 × 6 cm. Upper abdominal fullness was noted. Her haemogram, serum biochemistry, serum antibodies for HIV and HBsAg were negative with normal chest X-ray and cardiac evaluation. The serum CA-125 value was 142 U/ml. A CT scan of the abdomen and pelvis revealed multiple heterogeneously enhancing soft tissue masses within omentum, mesentry, perihepatic regions, and the pelvis. The ovaries were not seen separately. There was moderate ascites suggestive of malignant ovarian lesion with metastasis (Figures 1 and 2). Peritoneal fluid cytology was non-contributory.

**Figure 3:**
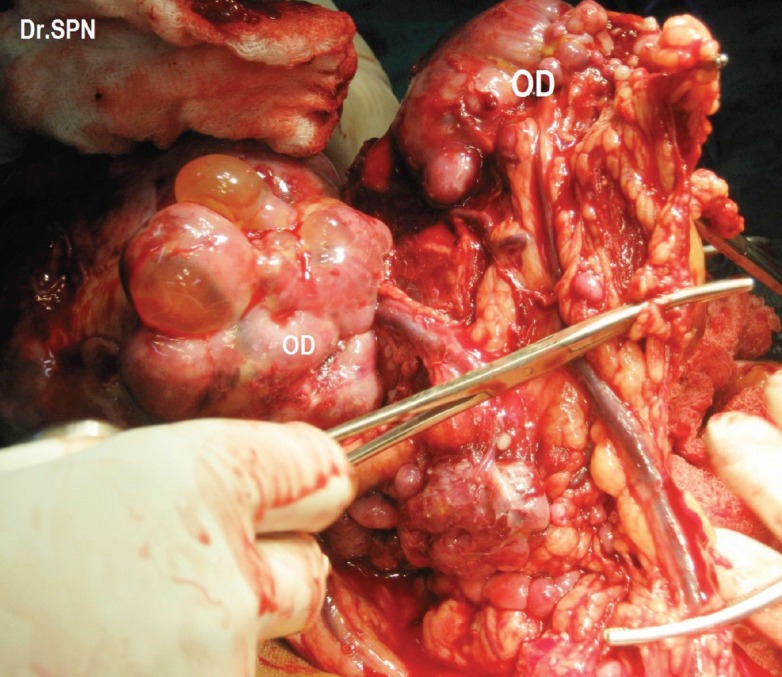
Intraoperative omental nodular metastatic deposits (OD).

**Figure 4:**
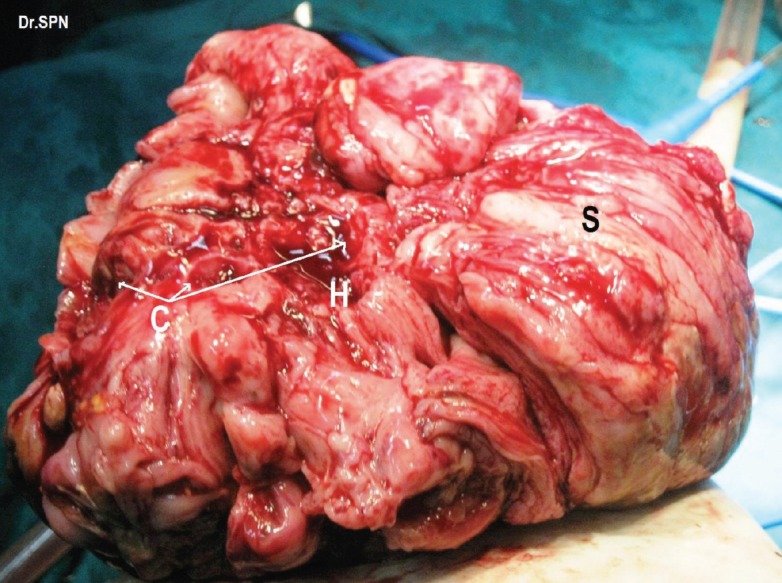
Right ovarian mass which appears nodular, breach in capsule, areas of haemorrhage (H), cystic spaces (C) and solid appearing areas (S).

**Figure 5:**
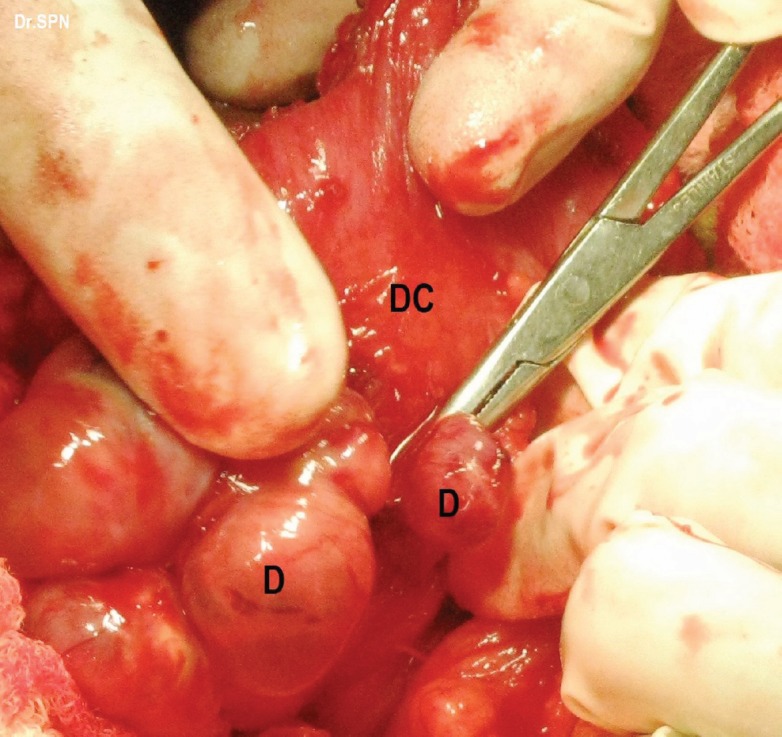
metastatic deposits (D) on the descending colon (DC).

**Figure 6:**
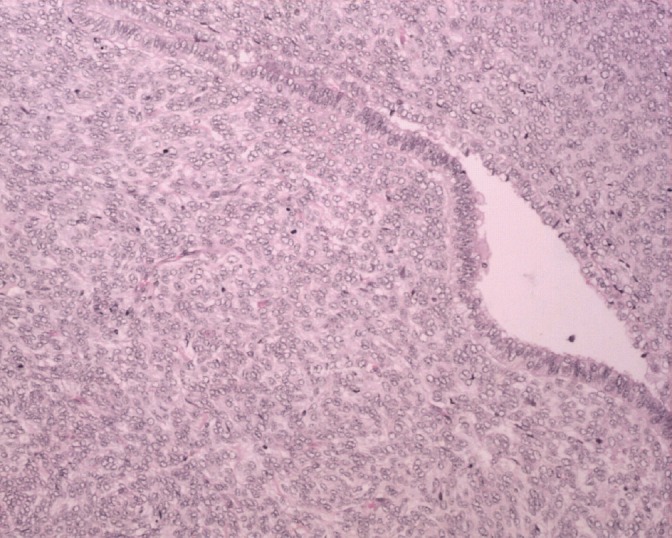
H&E x 20: biphasic neoplasm showing both benign epithelial component and sarcomatous mesenchymal component.

During laparotomy, 1 L of ascites was drained, a huge, vascular omental cake with multiple nodular deposits were seen (Figure 3). The right ovary measured 16 × 15 × 11 cm and was nodular and irregular with capsular breach (Figure 4). Metastatic deposits were seen on the mesentry, peritoneum, descending colon and bladder (Figure 5). On the table, frozen section of omentum showed short spindle cells with scanty hyalinized stroma with possibility of malignant stromal tumour of uterine or ovarian origin with metastasis to omentum was opined. Therefore, a complete tumour debulking, total omentectomy, left salphingo-oophorectomy, bilateral pelvic node dissection, peritonectomy, appendicectomy, excision of deposits on the bladder, bowel mesentry were performed to achieve optimal tumour load reduction. The post-operative period was uneventful. Histopathologic examination of the ovarian neoplasm and the peritoneal deposists revealed adenosarcoma of ovary (Figure 6).

**Figure 7:**
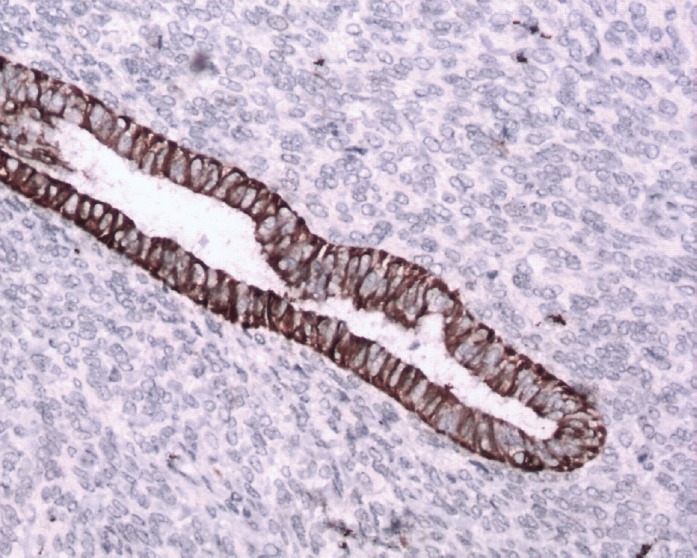
Immunostain CK7x20—epithelium is positive for CK7 (brown).

**Figure 8:**
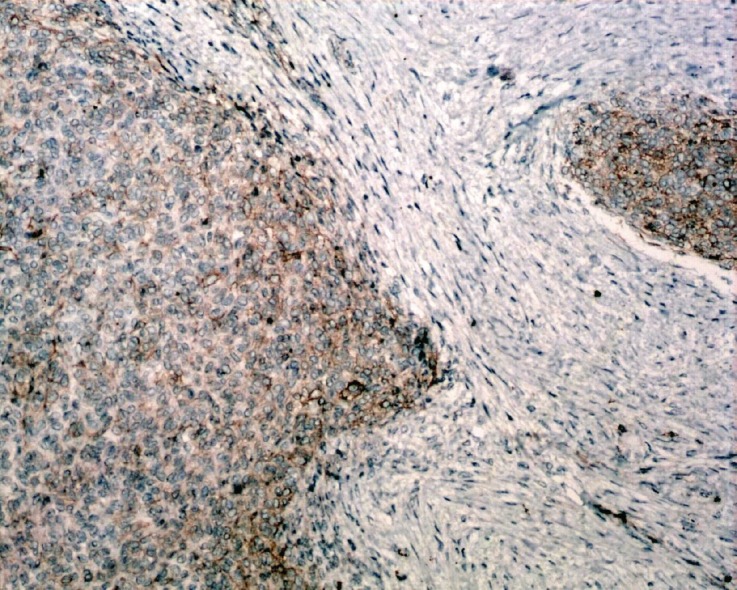
Immunostain, CD10x- Mesenchymal component is positivity for CD-10 (brown).

The IHC revealed neoplastic spindle cells which were positive for CD10 and negative for inhibin, C-kit, Calretiniun, SMA, S100, and Mic2, and the epithelium was positive for CK7 and EMA (Figures 7 and 8). She was allotted Stage IIIC.

She received five cycles of ifosamide with mesna and adriamycin every third week. She is on follow-up for more than 12 months, and there is no clinical, radiological, or biochemical evidence of recurrence.

## Discussion

Extrauterine Müllerian adenosarcomas are rare tumours. They can arise from the ovary, fallopian tubes, round ligament, pouch of Douglas, vagina, bladder, colon, and even peritoneum [2, 7, 9, 11].

The World Health Organization defines adenosarcoma as a biphasic tumour characterized by the proliferation of Müllerian epithelium of benign appearance or occasionally labelled atypical when absorbed in or covering a predominant sarcomatous stroma [12].

The present case has many clinical, therapeutic, and prognostic implications.

First, the adenosarcoma was arising from a previously normal ovary, which was salvaged for hormonal functions just 8 months earlier. These extrauterine Müllerian adenosarcomas occur at a younger age than their uterine counterparts and have more aggressive clinical behaviour because of invasion to adjacent pelvic organs at the time of diagnosis. Similar observations have been confirmed by many authors [4, 7, 9].

Second, homologous (pure) adenosarcomas arising from the ovary without associated endometriosis in either of the ovaries is very rare. Majority of the case reported have been associated with past, present, or concurrent evidence of endometriosis. The exact association is not clear in the literature as shared by many authors [4, 6, 8, 10, 11].

Third, ascitic fluid cytology may not be contributory, a frozen section may reveal the presence of malignant stromal tumour which needs further categorization and hence the importance of histopathology to delineate the benign and the malignant component of the tumour and immunohistochemistry to categorise the sarcomatous stromal component around which revolves the adjuvant therapy, prognosis and survival of the patients [10, 11]. To date, there is only one report of ascitic fluid cytology which could speculate neoplasm in an ovarian adenosarcoma [5].

Fourth, a raised CA-125 level being uncommon in adenosarcoma has been observed by authors as depicted in Table 1. The clinical implication of elevated CA-125 was first reported by Recinos *et al* [8] in an early stage disease. The present case was an advanced stage disease associated with elevated CA125 levels. She had optimal cytoreduction followed by 5 cycles of ifosamide with mesna and adriamycin every three weeks. Post-operative serum CA-125 level was 10 U/l. Serial serum CA-125 was used as a biochemical marker for follow-up. Associated fallacies of ascitic fluid cytology, frozen section and absence of endometriosis are reported for the first time in English medical literature (Table 1). She is on follow-up and there is no clinical, biochemical, or radiological evidence of recurrence for more than 12 months.

## Conclusions 

Multimodality treatment comprising optimal cytoreductive surgery followed by adjuvant ifosamide (mesna) and adriamycin-based chemotherapy may be an option for the treatment of these aggressive tumours. Follow-up with serial CA-125 values in advanced stage adenosarcoma of the ovary is a novel observation which needs to be researched.

## Figures and Tables

**Table 1.** Ovarian adenosarcoma. Characteristics of women, associated with elevated CA-125 (Normal-35 U/ml) levels, ascitic fluid cytology, and frozen section.

S no.	Author/ year	Age/parity/ presentation	Site of origin/ associated with endometriosis/ ascetic fluid cytology	Pre-treatment-CA-125-U/ml	Stage and homology/ frozen section	Treatment-surgical followed by adjuvant therapy	Post-treatment CA-125-U/ml
1	Inoue [3]/1995		Ovary/NA/NA	354	NA	NA/NA	17
2	Fukunaga [4]/1997	32, parous, abdominal pain	Ovary, Endometriosis/ NA	1100	Stage II, homologous/ not done	TAH + BSO + Oment.and PLND/ NA	NA*
3	Hirakawa [5]/2001	77/parity-NR/ abdominal pain	Ovary/NA/ suggestive of neoplasm	930	Stage II/III, NR/NA	NA/NA	7.4
4	Recinos [8]/2008	42/nulliparous/ incidental ovar-ian mass	Ovary, Opposite ovary/NA	1100	Stage IA, homologous/ not done	Extrafascial hysterectomy, BSO, Bilat. PLND, PO and PP biopsies + Medroxyprogester-one (MPA)	16
5	Present case/2012	40, parous, abdominal pain and distension	Ovary, no endometriosis/ only reactive mesothelial cells	142	Stage IIIC, homologous/ malignant stromal tumour-meta-static	O Rt. Ov. tumour debulking LSO, T LSO bil. PLN D excision of deposits on the descend-ing colon, and bladder. 5 cycles of Ifosamide(mesna) and adriamycin	10
*TAH* total abdominal hysterectomy, *BSO* bilateral salphingo-oophorectomy, *PLND* pelvic lymph node dissection, *TO* total omentectomy (supra and infra colic), *NA* not available, *PO* partial omentectomy, *PP* parietal peritoneal							
